# 2355: Phenotype and genotype in surviving relatives after sudden death in the young

**DOI:** 10.1017/cts.2017.103

**Published:** 2018-05-10

**Authors:** Gregory Webster, Rachael S. Olson, Zachary J. Schoppen, Nicholas Giancola, Alfred L. George

## Abstract

OBJECTIVES/SPECIFIC AIMS: Sudden death in the young (SDY) occurs in people between 1 and 40 years of age who do not have a known premortem risk factor for early death. Cardiovascular diseases account for the majority of causes of SDY. Sequencing of genes associated with congenital arrhythmia susceptibility and familial cardiomyopathy reveals pathogenic variants in 30% of postmortem cases (often called “molecular autopsy”). However, better data are needed to determine the prevalence of phenotype and genotype abnormalities in surviving relatives. METHODS/STUDY POPULATION: A retrospective cohort study was performed at a tertiary pediatric center including all subjects with a family history of SDY. Cases were identified using ICD-9 codes (798.1 or .9, V17.41, V17.49, V19.8, V61.07), search of cardiology databases, and by recursive identification of all family members of a subject. Phenotype data was independently reviewed by a pediatric cardiologist. Genotype results were available when obtained by the original treating physician. RESULTS/ANTICIPATED RESULTS: Cardiac evaluations were performed in 279 subjects from 175 families, of whom 117 subjects (42%) were first-degree relatives of the proband. Mean age of the subject at time of evaluation was 9 years (SD 5.9). Most probands were over 18 years at the time of SDY: 1–4 years of age (9%); 5–12 (5%); 13–17 (16%); 18–24 (18%); 25–40 (42%). A final diagnosis was determined in 55 families (20%), and a variant in a gene potentially causative of SDY was discovered in 20/55 (36%) of those families. Variants were classified as 50% pathogenic/likely pathogenic, 50% variants of unknown significance. Cardiac testing (ECG, echo, EST, signal averaged ECG, cardiac MRI, or EP study) was abnormal in 124/279 subjects (44%). Among those with abnormal studies, 57/124 (46%) were from a family where a final diagnosis could be determined (LQT 43%, HCM 21%, ARVC 4%, other cardiomyopathy 19%, WPW 5%, CPVT 2%). However, 67/279 of total subjects (24%) had at least 1 abnormal study and a final diagnosis was not determined in the family. DISCUSSION/SIGNIFICANCE OF IMPACT: An abnormal phenotype is common among relatives referred for cardiac evaluation after SDY. While testing identifies a family diagnosis in 20% of families, many patients have abnormal cardiac testing and no clear diagnosis can be made. An improved postmortem protocol for phenotype testing in relatives of a SDY victim and improved postmortem genetic testing may lead to a higher diagnosis rate and improved risk determination in surviving family members.

